# Presence of eimerid oocysts in faeces of a quarantined dog in Iceland is explained by coprophagic behaviour prior to its importation. Case report

**DOI:** 10.1186/s12917-020-02401-8

**Published:** 2020-06-15

**Authors:** Karl Skírnisson, Donald W. Duszynski

**Affiliations:** 1 Laboratory of Parasitology, Institute for Experimental Pathology, Keldur, University of Iceland, IS-112 Reykjavík, Iceland; 2 Department of Biology, University of New Mexico, 76 Homesteads Road, Placitas, NM 87043 USA

**Keywords:** *Eimeria canis*, *Eimeria magna*, *Eimeria stiedai*, Dog, Rabbit, Coprophagic behaviour, Case report

## Abstract

**Background:**

All dogs imported into Iceland must undergo mandatory quarantine in a special station before introduction into the country. A faecal sample is collected from the first stool passed by the dog in this station and subsequently examined for the presence of intestinal parasite stages.

**Case presentation:**

In May 2019 unsporulated oocysts were detected in faeces from a 7-year-old household dog that had been imported from Sweden. Most of the oocysts studied strongly resembled those of *Eimeria canis* Wenyon, 1923. As this species is not valid, the purpose of the present article was to identify the correct species and examine their possible origin. Studies confirmed the presence of two distinct unsporulated oocyst morphotypes in the faeces; measurements and photomicrographs confirmed their identification as *Eimeria magna* Pérard, 1925 and *Eimeria stiedai* (Lindemann, 1865) Kisskalt and Hartmann, 1907*,* both common parasites of European rabbits, *Oryctolagus cuniculus* (L., 1758). When the owner of the dog was questioned about the food administrated to the dog prior to its import to Iceland, it turned out that it had exclusively been fed dry dog food pellets. However, the owner also reported that on the morning prior to transportation to Iceland, the dog was allowed to move freely in a grassland area where rabbits are common and heaps of their faeces are present. Furthermore, the owner confirmed that the dog consumed rabbit faeces that morning.

**Conclusion:**

It is believed that this coprophagic behaviour can explain the detection of rabbit eimerids in the dog’s faeces, and that such behaviour must be taken into consideration by veterinarians and other diagnostic personnel when they detect atypical cysts or eggs during coprological examinations.

## Background

In the past century several authors have reported *Eimeria canis* Wenyon, 1923 to be a parasite of dogs, *Canis lupus familaris* L., 1758. In 1922 Brown and Stammers [1] examined 200 samples of dog faeces collected from the pavements of London for protozoa and other parasite stages. They noted “some coccidia-like bodies which did not develop under observation.” This stimulated Wenyon [2] to partially describe and name *E. canis* after having noticed a large, sporulated oocyst in dog scat found on the streets of London. In his initial description he remarked that in many respects the oocysts resembled a mixture of *E. stiedai* and *E. perforans* from the rabbit *Oryctolagus cuniculus*. A year later Nieschulz [3] also reported *E. canis* in dogs and Skidmore and McGrath [4] described it in a 3-month-old terrier from Nebraska, USA. Goodrich [5] thought that *E. canis* reported from dogs was a rabbit coccidium that dogs had consumed, with the oocysts passing through their gut unaltered. Later, Choquette and Gelinas [6] reported *E. canis* oocysts in faecal samples of 26 dogs in Montreal, and Bearup [7] said that he found oocysts of *E. canis* in Australian dingoes. Mimioğlu et al. [8] reported the species in a dog from Ankara, Turkey. Levine and Ivens [9] agreed with Goodrich [5] that, “It is far from certain that this is a valid species.” Skofitsch et al. [10] took measurements of *E. canis* oocysts “microscopically verified” from 6 dogs in Austria and presented photomicrographs of sporulated oocysts; however, Duszynski et al. [11] identified their oocysts as “clearly those of rabbit coccidia.” Nevertheless, *E. canis* continues to be reported in dogs, such as the account and commentary by Sudan et al. [12] in “non-descript dogs of Uttar Pradesh, India.”

The import of dogs to Iceland was prohibited, or restricted, during 1909–1989, as a measure to prevent the reintroduction of the already eradicated cestode, *Echinococcus granulosus*, that formerly caused widespread and serious human hydatidosis in Iceland [13, 14]. Since the ban was lifted in 1989, thousands of dogs have been imported into Iceland, originating from all continents of the world, except Antarctica [15]. According to regulations issued by the Icelandic Food and Veterinary Authority, all imported dogs are quarantined. Upon arrival in quarantine, the first faeces passed by each dog is examined for intestinal parasites. These samples are sent to the Laboratory of Parasitology, Institute for Experimental Pathology, University of Iceland, Keldur, where they are systematically examined for protozoan cysts and oocysts, and helminth larvae and eggs [15]. Each faecal sample is examined by two methods: 1.) The formalin-ethyl acetate sedimentation technique (FEAST) is used to concentrate protozoan cysts, coccidian oocysts, helminth eggs and larvae [16, 17] using the *Faecal Parasite Concentrator* (FPC®) Kit from Evergreen Scientific. And 2.) A modified Baermann technique, based on the description of Henriksen [18] is applied. Preferably 25–30 g of fresh faeces is wrapped in a double (or in the case of soft stool into a triple) layer of cheesecloth (absorbent gauze in rolls, Mullro®) and submerged into a 375 ml conical “Baermann” glass filled with 300 ml of tap water. The sample is kept immersed in the water for 6–24 h (usually overnight) at room temperature (approximately 22 °C). Upon examination all sediment from the bottom of the glass is pipetted onto one or more microscope slides, mixed there with a drop of iodine, and screened at 62.5X magnification in a light microscope. Mainly nematode larvae (e.g. *Strongyloides stercoralis* or *Angiostrongylus vasorum*) are detected with this method [19] but sometimes also helminth eggs and coccidian oocysts that fall to the bottom of the Baermann glass. Usually, only this first sample is examined from each dog but sometimes, as in the present case, an additional sample is collected and analysed.

From January 1989 to May 2019, faecal samples from 4171 imported dogs were screened for parasites. Three coccidians of the genus *Cystoisospora* were identified in a total of 29 dogs (0.7%); *C. ohioensis* was detected in 13/4171 (0.3%), *C. canis* in 9/4171 (0.2%), and *C. burrowsi* in 7/4171 (0.2%). All three species are known to occur in the native dog population in Iceland [15]. A representative of the genus *Eimeria* has only been detected once in such a faecal sample (1/4171 (0.02%)), that is the case described here.

## Case presentation

According to Icelandic regulations imported dogs must be under quarantine for 4 weeks after their arrival and a sample from the first faeces passed by the dog in the quarantine station has to be examined for the presence of internal parasites. In May 2019 a 7-year-old female dog (Coton De Tulear) that had lived in a suburb of Stockholm in Sweden was imported to a quarantine station in Iceland. Parasitological examinations revealed previously unknown coccidian oocysts. Morphologically, some of the oocysts clearly resembled a line drawing of *E. canis* published by Levine and Ivens [9]. As eimeriid coccidians had not previously been detected in dog faeces in Iceland, this finding encouraged a study of their morphology to identify the species involved and examine their possible origin.

### Description of the eimeriid oocysts

**Fig. 1:**
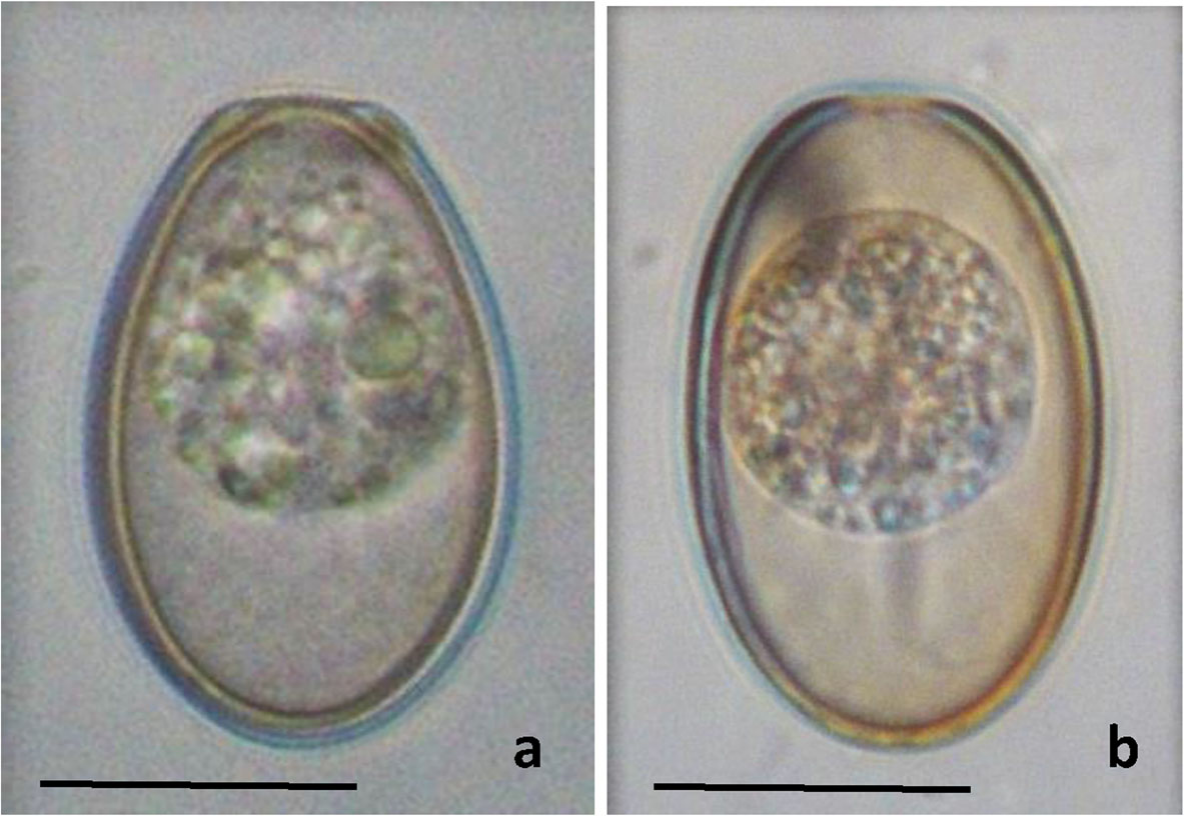
Nomarski interference contrast photomicrograph of unsporulated *Eimeria magna***a** and *Eimeria stiedai***b** oocysts (parasites of European rabbits *O. cuniculus*) detected in the faeces of a coprophagic dog after being transferred from Sweden to a quarantine station in Iceland. *Bar* = 20 μm

Using the Baermann technique, 6 large unsporulated oocysts with a flat mircopyle end were detected, and some of them appeared to have a prominent thickening at the micropyle end (Fig. 1a). A quick comparison of this previously unknown dog coccidian showed certain similarities to a line drawing of *E. canis* by Levine and Ivens [9], as previously mentioned.

Morphological examination at a higher magnification (1000X) and subsequent photography of formaldehyde-fixed oocysts revealed the presence of two distinct, unsporulated oocyst forms (Figs. 1a, b). According to line drawings and identification keys [20–23] the oocysts strongly resembled the morphological descriptions of *E. magna* and *E. stiedai*, respectively, coccidians that are both known to infect the European rabbit, *O. cuniculus*. Oocysts of both species are similar in size and shape. Those of *E. magna* are ovoidal and have a prominent micropyle with a thickened collar around it; these oocysts measured, L × W (*n* = 9) 38.9 × 23.3 μm, L/W ratio, 1.7; a large oocyst residuum is present. Oocysts identified as those of *E. stiedai* were elongate-ellipsoidal with a narrow, ill-defined micropyle; these oocysts were, L × W (*n* = 3) 38.5 × 23.0 μm, L/W ratio, 1.7; a minute oocyst residuum is present. Both species are common parasites of rabbits and have a worldwide geographical distribution.

A second faecal sample taken 3 days after the arrival of the dog in the quarantine station in Iceland, examined by the FEAST method, appeared to be free of coccidian oocysts.

### Food consumption by the dog

After having identified rabbit eimeriids from the first faecal sample passed by the dog following arrival in Iceland the owner was contacted and asked what the dog had been fed prior to its arrival in Iceland. Only dry food pellets had been given, rabbit products were not fed to the dog. However, knowing that dogs exhibit coprophagic behaviour [24] this observation stimulated us to ask about the activity of the dog before it was prepared for its transport to Iceland. The owner described a situation that could explain the presence of rabbit eimeriids in its faeces. On the morning prior to travel, the dog was allowed to move freely in a grassland area where rabbits are common and heaps of rabbit faeces were present. Moreover, this particular morning the dog was observed consuming rabbit faeces in the area. This coprophagic behaviour of the dog was well-known to its owner and had frequently been observed before. Interestingly, none of the examined oocysts (*n* = 12) had sporulated, indicating that the dog had selected fresh rabbit faeces to consume.

## Discussion and conclusions

Frenkel and Parker [24] published an interesting paper in which they examined the apparent role of dogs in the transmission of *Toxoplasma* gondii, a parasite of cats that can infect humans, and surveyed the frequency with which dogs consumed and rolled in faeces in Santa Fe, New Mexico, USA. The part of their study that is relevant here is that 29/52 (55.7%) of the dogs they studied were documented to eat faeces of other animals. Prior to their paper [24], the veterinary literature had considered coprophagy to be abnormal behaviour [25]. However, we now know that coprophagy is a widespread behaviour among small- and medium-sized mammals, including dogs, to help them rebalance their intestinal microbiome and meet their body’s nutritional requirements. Such observations have opened an entirely new discipline for study in animal health. Early on, for example, Hörnicke and Björnhag [26] suggested coprophagy can help improve the digestive function of herbivorous animals because many nutrients and food fragments can be digested and absorbed again by eating faeces. In addition to nutritional benefits, coprophagy also may help some mammals retain their needed gut microbial diversity and function, which has downstream physiological effects to maintain energy balance and cognitive function. Bo et al. [27] reported that when certain mammals did not practice coprophagy, it decreased diversity of their gut microbiota, and altered abundances of specific microbial taxa. Thus, there seems a clear relationship between coprophagy and interactions between the gut microbiota and energy metabolism [28] and a wide range of compounds generated by gut microbiota are known to have direct or indirect effects on neurological function [29]. Such ideas initially generated from field observations and simple laboratory models have spawned an entire new approach to understanding evolution with the moniker, the hologenome concept [see [30].

Wenyon [2] remarked that the oocysts he originally described from a dog, in many respects resembled a mixture of *E. stiedai* and *Eimeria perforans* from rabbits. Goodrich [5] thought that *E. canis* reported from dogs was a rabbit coccidium that dogs had eaten, with the oocysts passing through their gut unaltered. Frenkel and Parker [24] clearly demonstrated that dogs commonly eat the faeces of many other animals. Duszynski et al. [11] identified oocysts presented by Skofitsch et al. [10] as rabbit coccidia and these authors also agreed with Levine and Ivens [9] that *E. canis* is not a valid species. They included the species description in their recent book on *The Biology and Identification of the Coccidia (Apicomplexa) of Carnivores of the World* only for historical purpose and are convinced that this “species” along with all other *Eimeria* species described from dogs are oocysts from prey animals [11].

In conclusion, we have documented coprophagic behaviour by a dog that explains the presence of *Eimeria* oocysts from rabbits found in the dog’s faeces. This emphasizes the importance of studying the epidemiology of every infection. Coprophagy is common in many domestic and wild mammals and in some it may be critical in maintaining the nutritional benefits of their complex gut microbiome. Such behaviour shouldn’t be overlooked, and it should be taken into consideration by veterinarians and other diagnostic personnel when they detect atypical cysts or eggs during coprological examinations so they don’t describe exotic infections or new parasite species that don’t actually exist.

## Data Availability

The datasets used and analysed during the current study are available from the corresponding author on reasonable request.

## Abbreviations

e.g.: For example
FEAST: Formalin-Ethyl Acetate Sedimentation Technique
FPC: Faecal Parasite Concentrator
L: Length
W: Width
n: Number
μm: Micrometer
